# Neuropalliative care: new perspectives of intensive care

**DOI:** 10.5935/0103-507X.20210016

**Published:** 2021

**Authors:** Erica Regina Ribeiro Sady, Lígia Maria Coscrato Junqueira Silva, Viviane Cordeiro Veiga, Salomón Soriano Ordinola Rojas

**Affiliations:** 1 BP - A Beneficência Portuguesa de São Paulo - São Paulo (SP), Brazil.

**Keywords:** Palliative care, Nervous system diseases/surgery, Intensive care units, Cuidados paliativos, Doenças do sistema nervoso/cirurgia, Unidades de terapia intensiva

## Abstract

Neurological diseases are estimated to affect 1 billion people worldwide and are the cause of one in 10 deaths. In Brazil, they are responsible for approximately 14% of clinical admissions to intensive care units, 9% of elective neurosurgeries and 14% of emergency neurosurgeries. Many of these conditions are incurable, result in reduced life expectancy and quality of life and increased dependence, and are associated with symptoms that are likely to cause suffering, which justifies the integration of palliative care into usual care. In addition, factors unique to acute neurological injuries, such as their catastrophic clinical presentation, complex and uncertain prognosis, associated communication difficulties and issues related to quality of life, require a specific approach, which has recently been termed "neuropalliative care". Although the topic is relevant and current, it is still little discussed, and much of what is known about palliative care in this context is extrapolated from approaches used under other conditions. Therefore, the objective of this study was to conduct a narrative literature review to identify the challenges of applying the palliative care approach in the care of neurocritically ill patients, with a focus on three groups: neurocritically ill patients, families and intensive care teams. This review identified that in intensive care, the main demands for palliative care are for prognostic definitions and care planning. Training in primary palliative care and improving communication were also needs identified by intensivists and families, respectively. In contrast with what has been found under other conditions, the management of symptoms was not indicated as a complex issue, although it is still relevant.

## Abstract (pt)

Estima-se que as doenças neurológicas acometam 1 bilhão de pessoas no mundo e sejam causa de uma a cada dez mortes. No Brasil, são responsáveis por, aproximadamente, 14% das internações clínicas em unidades de terapia intensiva, 9% das neurocirurgias eletivas e 14% em urgência. Muitas dessas condições são incuráveis, implicam em reduzida expectativa e qualidade de vida e maior dependência, além de estarem associadas a sintomas que predispõem ao sofrimento, o que justifica a integração dos cuidados paliativos aos usuais. Além disso, fatores peculiares às injúrias neurológicas agudas, como apresentação clínica catastrófica, prognóstico complexo e incerto, dificuldade de comunicação e questões relacionadas à qualidade de vida exigem uma abordagem específica, recentemente denominada "cuidados neuropaliativos". Assim, embora relevante e atual, o tema ainda é pouco discutido e muito do que se conhece sobre cuidados paliativos nesse contexto são extrapolações sobre abordagens em outras condições. Por isso, foi objetivo deste estudo realizar uma revisão narrativa da literatura, a fim de identificar os desafios da abordagem dos cuidados paliativos na assistência aos pacientes neurocríticos, com foco em três núcleos temáticos: pacientes neurocríticos, familiares e equipes de terapia intensiva. Esta revisão identificou que, no intensivismo, as principais demandas de cuidados paliativos são para definições prognósticas e planejamento de cuidados. A necessidade de treinamento em cuidados paliativos primários e de aprimoramento da comunicação também foi uma necessidade identificada por intensivistas e pelas famílias, respectivamente. De modo diferente do que se dá em outras condições, o manejo de sintomas, apesar de relevante, não foi indicado como questão complexa.

## INTRODUCTION

Neurological diseases are estimated to affect approximately 1 billion people worldwide and are the cause of one in 10 deaths.^(1)^ Many of these conditions are incurable, result in reduced life expectancy and quality of life and greater dependence, and are associated with symptoms that predispose patients to suffering, which justifies the integration of palliative care (PC) into usual care.^(1-3)^

In Brazil, recent data show that neurocritical diseases are responsible for approximately 14% of hospitalizations in intensive care units (ICUs), 9% of admissions for elective neurosurgery and 14% of admissions for emergency surgery.^(4)^ However, although the objective of intensive care is to recover the organ function of individuals at risk of imminent death and/or in a fragile clinical condition and who will potentially benefit from therapeutic interventions, it is estimated that 14% - 20% of critically ill patients have an indication for PC, with projections that this number will double by 2030.^(5-7)^

Although this topic is relevant and current, it is still little discussed, and much of what is known about PC in this context is extrapolated from approaches applied under other conditions.

The objective of this study was to identify, through a narrative literature review, the particularities and challenges of the PC approach for the care of neurocritically ill patients, with a focus on three groups: patients, families and intensive care teams. A brief description of the reviewed studies is shown in table 1 of Appendix 1.

**Table 1:** Included studies

Authors	Thematic axes		
	Patients	Family	ICU staff
Brizzi et al.^(2)^	x		
Tran et al.^(8)^	x	x	x
Geurts et al.^(9)^	x	x	x
Frontera et al.^(10)^	x		
Bar et al.^(11)^	x		x
Creutzfeldt et al.^(12)^	x		
Cai et al.^(13)^	x	x	
Owens et al.^(14)^	x		
Tabibian et al.^(15)^	x		
Souter et al.^(16)^	x	x	x
Kross et al.^(17)^	x		x
Rubin et al.^(18)^	x	x	
Creutzfeldt et al.^(19)^	x		
Adil et al.^(20)^	x	x	x
Creutzfeldt et al.^(21)^	x		x
Knies et al.^(22)^	x		
Schaller et al.^(23)^	x		x
Torbey et al.^(24)^	x		
Smith et al.^(25)^	x		
Davidson et al.^(26)^	x		
World Health Organization^(27)^	x		
Creutzfeldt et al.^(28)^		x	
Khan et al.^(29)^		x	
Schutz et al.^(30)^		x	
Muehlschlegel et al.^(31)^		x	
Trevick et al.^(32)^		x	
Steigleder et al.^(33)^		x	
Miranda et al.^(34)^			x
Christakis et al.^(35)^			x
Nelson et al.^(36)^			x
Bluck et al.^(37)^			

ICU - intensive care unit.

### Neuropalliative care

**Figure 1:**
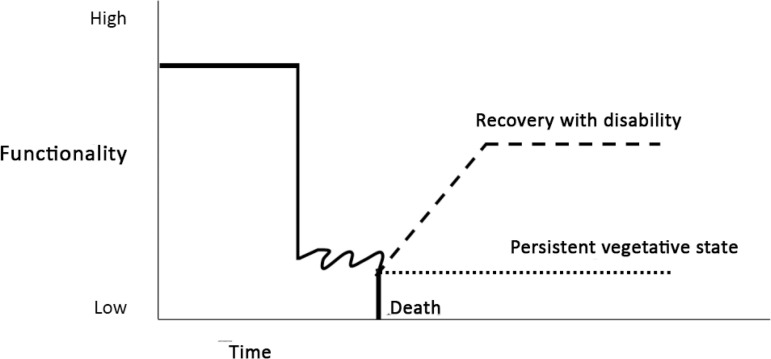
Trajectory of neurocritical diseases.

Acute neurological diseases affect essential functions related to cognition, communication and identity, among other domains.^(8)^ In the context of intensive care, this impact is even more evident, especially because these injuries cause an abrupt and drastic change in the course of the lives of these patients and their families/caregivers and their progression is uncertain (Figure 1 of the Appendix 1).^(8,9)^

Hypothetical clinical trajectory after acute neurological injury. Note the abrupt decline in functionality after the event. The possible outcomes are death, survival in a persistent vegetative state or survival and recovery with associated disability.

Source: adapted from Frontera JA, Curtis JR, Nelson JE, Campbell M, Gabriel M, Mosenthal AC, Mulkerin C, Puntillo KA, Ray DE, Bassett R, Boss RD, Lustbader DR, Brasel KJ, Weiss SP, Weissman DE; Improving Palliative Care in the ICU Project Advisory Board. Integrating Palliative Care into the Care of Neurocritically Ill Patients: A Report from the Improving Palliative Care in the ICU Project Advisory Board and the Center to Advance Palliative Care. Crit Care Med. 2015;43(9):1964-77.^(10)^

These conditions, which are potential sources of suffering, highlight the multiple challenges that PC faces in the neurointensive care context. Furthermore, given their particularities, these challenges require their own approach, which was recently termed "neuropalliative care".^(2,10)^

### Neurocritically ill patients

The literature indicates that a wide variety of neurological patients can benefit from PC.^(2)^ Brizzi et al. propose a classification of these patients into four categories according to the progression of neurological disease to assist in the identification of specific needs: (1) patients with rapid or (2) prolonged decline, (3) episodic decline and (4) acute decline and uncertain recovery.^(2)^ In cases of acute decline, for example, the demand for symptom management may be high, while for those with prolonged decline, eventual caregiver burnout may be a more evident need.^(2)^

The unique profile of neurocritically ill patients should be highlighted, as indicated by the responses of intensivists from the Neurocritical Care Society (NCS)^(11)^ to a questionnaire applied to evaluate their perceptions and preferences about the integration of PC into ICUs.^(11)^ The following factors were identified as particular to this population: catastrophic clinical presentations; a complex and uncertain prognosis; difficulty with communication due to neurological injuries; and issues related to disabilities and postinjury quality of life.^(11)^

Given this, it is evident that the PC approach for these patients differs from that for patients with other conditions, such as cancer. While in this condition, the demand for symptom management is common, among neurocritically ill patients, prognostic evaluations demand greater attention due to their complexity.^(8-10,12-18)^

However, the prognostic models available for use in neuropalliative care have limitations. Among other shortcomings, many do not have good accuracy to support end-of-life decisions, present low external validity and limited sensitivity, or were developed for use in the first days after neurological injury and are less applicable for chronic critical cases.^(16,18-20)^

In addition, the fact that these models focus on the outcomes "death" and "functional disability" was also considered limiting, especially because supportive interventions in neuropalliative care tend to prolong survival time, despite greater dependence and a lower quality of life.^(9,10,12-14)^ Thus, residual quality of life and the possibility of functional recovery seem to be more relevant outcomes for patients and families.^(8-10)^

Rapid neurological deterioration, which often requires immediate clinical and/or neurosurgical intervention, and the consequent loss of the patient's ability to manifest his or her will are also factors particular to this population.^(3,17,21)^ Bar et al. highlighted that neuropsychological and behavioral changes, including impulsivity, loss of empathy, apathy, depression and obsessive-compulsive disorder, also make the management of these patients challenging.^(11,16,22)^

This is worrisome because decision-making ability is considered a vital criterion in the validation of consent for health treatments. However, this requires an understanding and critical appreciation of the situation and the potential consequences of one's choices, in addition to the ability to make decisions in the first place and to communicate these decisions to third parties.^(9,16,23)^

Therefore, efforts to identify neurocritically ill patients who can benefit from the integration of PC with usual care is essential.^(12)^ For this purpose, Creutzfeldt et al. developed a screening tool, in the form of a checklist, for daily use in the ICU.^(19)^

The instrument consists of questions with dichotomous answer choices: "Does the patient have distressing physical and/or psychological symptoms?"; "Are there specific social/support needs for patient and/or family?"; "Have goals of care been identified and are treatment options matched with patient-centered goals?"; and "Are there disagreements within teams, family or between those?" An affirmative answer to any of these questions was considered indicative of the need for primary PC.^(19)^

In the Creutzfeldt et al. study, 62% of patients required PC, and the most frequently mentioned needs were for social support and care planning, which reaffirms the specificities of this group of patients.^(19)^ In addition, the use of this tool resulted in a higher frequency of cross-consultation with specialized social work and PC services, in addition to more conferences between teams and families.^(19)^

However, although the literature suggests that the early introduction of PC is related to a reduction in costs secondary to the length of ICU stay, some studies warn that caution is necessary.^(24,25)^ It is argued that "pessimistic prognoses" result in a less intervention-based approach, and this could contribute to an unfavorable clinical progression in the manner of a self-fulfilling prophecy.^(9,25)^ However, in our analysis, this outcome is the result of a conflict between concepts that must be clarified.

In fact, it is known that in the acute phase, neurocritically ill patients are exposed to factors that may confound an adequate neurological evaluation, such as the effects of neurodepressants and therapeutic hypothermia.^(24)^ In addition, it should be considered that the care scenario itself and the expertise of teams seem to be factors that can influence outcomes.^(24)^

Therefore, in cases of acute neurological injury, it is rational to recommend caution in limiting support measures and to ensure an adequate observation period before making decisions.^(16,24)^ In addition, it is suggested that, ideally, patients with acute neurological injury be transferred to specialized centers.^(24)^ However, it is crucial to understand that instituting PC is not equivalent to limiting treatment, which represents an outdated and inadequate view of this approach.^(26)^

According to the World Health Organization (WHO), PC is a team-based approach aimed at improving the quality of life of individuals - adults and children - who face life-threatening diseases, as well as that of their families and/or caregivers.^(27)^ It aims to prevent and relieve suffering through early identification, evaluation and adequate management of pain and other physical, psychosocial and/or spiritual problems related to any condition.^(27)^ Therefore, it aims to encourage patients to live as actively as possible in all phases, from diagnosis to death, which includes comforting family members during bereavement.^(27)^

Given the prognostic uncertainty in this scenario, caution should be exercised when deciding to limit potentially inappropriate treatments. However, this same uncertainty regarding prognoses, superimposed on other potential sources of suffering (functional limitation, increased dependence and reduced quality of life secondary to neurological injury), is the reason PC should be instituted early, ideally in integration with the usual intensive care.

### Families

Disability is an outcome often associated with neurological injury, and communication limitation is one of the most frequently reported disabilities.^(13,14,22)^ In such cases, families become involved in shared therapeutic decisions; there involvement is also important because it is unquestionable that in most cases, due to their coexistence and bond with the patient, they are the parties with the best ability to understand the patients' preferences.^(16,18,28)^

However, considering that even the best prognostic estimates for neurological injuries are accompanied by a wide margin of uncertainty, therapeutic decisions tend to be postponed.^(9)^ Nonetheless, it is recommended that intensivists not avoid frank, prognostic discussions with family members because unrealistic expectations may result in the expenditure of resources without justifiable benefits.^(9)^

However, this is not a simple task; it is common for family members to be unaware of the patients' preferences and values, and dysfunctional relationships may exist between patients and family members.^(9)^ In addition, the scenario of acute neurological injury exposes family members to stress, which, together with unpreparedness, hesitancy (or excessive optimism) and uncertainties regarding the future, impacts their competence to make wise decisions.^(9,13,16,20)^

Tran et al., for example, found that only a small number of neurocritically ill patients had expressed their care preferences prior to their injury.^(8)^ In the cases in which the patients had done so, their relatives were reluctant to comply with the patient's directives because they believed that the damage resulting from the injury would be temporary and that certain interventions would be justified.^(8)^

Those authors, as well as Khan et al., suggested an approach that consisted of asking family members to describe who the patient was and what their values were; "independence" and "ability to interact" were the most cited qualities.^(8,29)^ Next, the family members' level of understanding, perceptions and beliefs about the clinical condition were investigated.^(8)^

After that, the families were informed about the treatment options available and their advantages and disadvantages.^(8)^ According to the authors, this helped resolve conflicts when there was disagreement about care goals and/or communication difficulties among the parties.^(8)^

Furthermore, Schutz et al. identified that, in a scenario of uncertainty, families tend to connect with intensivists based on feelings of confidence and support and on the hope that these professionals transmitted.^(30)^ In addition, families valued the perception that the patient was treated as a person in the care process and the that teams considered the needs of family members equally relevant as aspects of compassionate and attentive care.^(30)^

A need for information was also identified. "Is there hope?" was one of the most important questions families wanted to have answered; another was "What is the chance that the patient will walk/talk/take care of themselves again?"^(30)^

The literature indicates that depressive symptoms and posttraumatic stress disorder are not uncommon among families of neurocritically ill patients.^(13,28,31,32)^ Therefore, Cai et al. suggest that the first meeting between family members and the team should occur in the first 24 - 48 hours after ICU admission; although this is early for prognostic predictions, early contact is important for establishing trust and alleviating stress.^(13)^ In these meetings, assistive methods, such as instructional videos, can be used to facilitate communication.^(13,32)^

While a high degree of empathy and respect for family members should be maintained, as recommended by the NCS, when families have unrealistic expectations about the patient's prognostic progression, they should be redirected toward possible short-term goals.^(13,16,20,31,33)^

### Intensive care teams

Although intensivists' perceptions of PC and the challenges of integrating this approach with the usual care in their practice are relevant, they are minimally discussed.

Kross et al. evaluated the extent to which medical specialty might influence indications for PC.^(17)^ In that study, PC was offered less frequently when patients were under the care of surgeons (12%), and this rate was even lower in the neurosurgery (6%) and neurology (3%) specialties.^(17)^ A possible explanation is found in the study by Schaller and Kessler, in which most neurosurgeons indicated that end-of-life decisions, which included refusing neurosurgical interventions, limiting or suspending life support and following nonresuscitation orders, were "difficult" or "very difficult".^(23)^

Inadequate training in PC was also a factor frequently reported in the literature. This issue presents special challenges because of an unintentional lack of skills to address complex issues such as delivering bad news and discussing end-of-life care and limitation of support, which require a high degree of interventionist management, even when the prognosis is unfavorable.^(20,34)^

One study indicated neurosurgery residents do not have sufficient training to consider themselves able to make decisions about limiting life support.^(34)^ In that study, neurosurgery residents underwent an assessment of their prognostic ability in hypothetical scenarios in which there was a high degree of uncertainty regarding whether interventions would benefit survival, functionality and quality of life.^(34)^ Although the majority of the residents performed well, only a small percentage had actually been trained or received feedback from their supervisors about their own performance, which the residents indicated was a cause of "moral stress".^(34)^

Despite these findings, in one survey, the majority of neurointensivists in the NCS said they believed that PC added value to intensive care and positively impacted patient outcomes.^(11)^ In that study, the need for care planning, including decisions related to the withdrawal of support, was the most frequent reason for consulting the specialized PC team.^(11)^ However, given the conceptual definition of PC,^(27)^ this should not be the only time that a PC approach should be considered. As discussed, intensivists consider the prognostic evaluation of neurocritically ill patients a challenge, despite the availability of prognostic tools, and this is still the main factor that motivates cross-consultation with the PC team.^(11)^

There is a known tendency toward optimism in prognostic predictions for critically ill patients, and the accuracy of these predictions seems to be further reduced when there is a strong patient-family bond.^(35)^ In contrast, when professionals are more experienced, prognostic accuracy is improved.^(35)^ Thus, the combination of the clinical judgment of experienced professionals and the use of prognostic tools seems to be the best strategy in a scenario of uncertainty.^(9)^

Another challenge highlighted by intensivists refers to decision making. These professionals are concerned with issues related not only to the survival of neurocritically ill patients but also to their quality of life after their neurological insult.^(11,21)^ This is because, although neurocritically ill patients are not always terminally ill, their acquired disabilities may result in an "unacceptable" quality of life.^(11)^

Despite this, few professionals are aware of the patients' values when making decisions. In the study by Miranda et al., when asked about real cases, less than 60% of neurosurgery residents said they knew that the care offered was compatible with the patient's values.^(34)^ In other cases, interventions were performed despite the lack of information about patient preferences, which confirms that skills related to end-of-life discussions are necessary, including for interventionists.^(35)^

Another dilemma relates to how to integrate PC teams into cases. Despite its undeniable relevance in complex cases, there is still no consensus regarding the most appropriate way to integrate PC and intensive care.^(11)^ Some strategies include cross-consultations or notifications based on clinical decision or objective criteria, or even electronically, when a patient meets the criteria for indication.^(11)^ Another option is the inclusion of PC workers in the ICU as part of the team.^(11)^

Having overcome this dilemma, who would be responsible for providing patient prognoses? Tran et al. suggest that this should be done by teams with enhanced technical competence in the management of neurological injury and a greater relational bond with the patient and family.^(8)^

This would require education and training in an integrative approach that aims not to replace the consultative model in cases that require the expertise of PC specialists but rather to train professionals who have greater contact with neurocritically ill patients in the early identification of the need for PC and the assertive implementation of PC approaches.^(36,37)^ Additionally, the involvement of other services, such as social work, psychology and spiritual care, is recommended.^(16)^

### Strategies for promoting neuropalliative care in intensive care

Improving the ability of intensivists to provide primary PC to neurocritically ill patients is a quality goal that must be achieved.^(9,17)^ For this purpose, it is necessary to teach skills to nonspecialists, as recommended by the Accreditation Council for Graduate Medical Education (ACGME).^(35,38)^

Strategies to overcome cultural barriers are also recommended. The main barrier in this regard is the belief that PC should be introduced only at the end of life, in scenarios with greater prognostic certainty or likely unfavorable outcomes, and/or when the decision to withdraw life support has been made.^(14,20,33,37)^

A proactive assessment facilitated by the use of instruments (checklists, for example) encourages teams and families to discuss important, albeit sometimes difficult, subjects while reducing the influence of cognitive bias.^(19,27)^ Such assessments are not intended to increase referrals for PC but to develop professionals' qualifications to recommend it in a more judicious manner.^(19)^

There is also a need to develop and/or translate and validate instruments for the Brazilian population. Ideally, such instruments should be short and easy to understand to encourage the adherence of non-PC specialists in a scenario with multiple demands, such as intensive care.

Finally, a strategic five-stage approach to integrating PC was proposed by Geurts et al.^(9)^:

(1) Collect evidence: perform prognostic evaluation and discuss the risks and benefits of possible interventions, including those resulting from the decision not to intervene.
(2) Share information: inform the family about the disease, treatment options and prognosis with or without interventions; consult the family about the patient's values and preferences.
(3) Critical appraisal: the team and family should critically appraise the clinical scenario and the risks inherent to each decision.
(4) Recommendations and decisions: intensivists should then integrate and synthesize the information and make their recommendations, aiming to reach a shared decision with the family.
(5) Evaluation and follow-up: professional performance should be assessed, and follow-up should be maintained.

### Future

The integration of PC with traditional approaches is a reality; furthermore, it is the right of patients and families for whom the approach is indicated. Thus, Creutzfeldt et al.,^(1)^ in a summit held during the 2017 American Academy of Neurology meeting, proposed some lines of action to stimulate the development of neuropsychological care in clinical, research and educational settings that were also recommended by other studies included in this review:^(3,9,11,17)^

- Develop and implement effective models to integrate PC into neurological care.
- Develop and implement quality indicators to evaluate and compare the effectiveness of palliative approaches.
- Improve the use of resources to promote patient-centered care.
- Improve access to PC and update the PC criteria for neurological disorders.
- Reduce the stigma of PC.
- Increase access to education in neuropalliative care for all professionals.
- Create a standardized curriculum for education in primary PC for neurologists.
- Incorporate PC training into residency/specialization programs.
- Develop epidemiological research and interventions (pharmacological, technological and behavioral) with accurate evaluations and the involvement of the patient and family in care to promote changes in practices and knowledge dissemination.

## FINAL CONSIDERATIONS

This review identified that in intensive care, the main demands for PC relate to prognostic definitions and care planning. The need for training in primary PC and communication improvement were also identified by intensivists and families, respectively. In contrast with PC for other conditions, symptom management, was not indicated as a complex issue in PC for neurocritically ill patients, although it was still relevant.

Thus, the relevance of PC for neurocritically ill patients, their families and health care teams is evident as it aims to improve intensive care through an integrative approach that is compatible with the values and experiences of autonomy and dignity and with the wishes of patients and their families.
